# The development of a questionnaire to assess leisure time screen-based media use and its proximal correlates in children (SCREENS-Q)

**DOI:** 10.1186/s12889-020-08810-6

**Published:** 2020-05-12

**Authors:** Heidi Klakk, Christian Tolstrup Wester, Line Grønholt Olesen, Martin Gillies Rasmussen, Peter Lund Kristensen, Jesper Pedersen, Anders Grøntved

**Affiliations:** 1 Research Unit for Exercise Epidemiology, Centre of Research in Childhood Health, Department of Sports Science and Clinical Biomechanics, University of Southern Denmark, 5230 Odense M, Denmark; 2 Research Center for Applied Health Science, University College Lillebælt, 5230 Odense M, Denmark

**Keywords:** Screen-media use, Children, Questionnaire, Correlates

## Abstract

**Background:**

The screen-media landscape has changed drastically during the last decade with wide-scale ownership and use of new portable touchscreen-based devices plausibly causing changes in the volume of screen media use and the way children and young people entertain themselves and communicate with friends and family members. This rapid development is not sufficiently mirrored in available tools for measuring children’s screen media use. The aim of this study was to develop and evaluate a parent-reported standardized questionnaire to assess 6–10-year old children’s multiple screen media use and habits, their screen media environment, and its plausible proximal correlates based on a suggested socio-ecological model.

**Methods:**

An iterative process was conducted developing the SCREENS questionnaire. Informed by the literature, media experts and end-users, a conceptual framework was made to guide the development of the questionnaire. Parents and media experts evaluated face and content validity. Pilot and field testing in the target group was conducted to assess test-retest reliability using Kappa statistics and intraclass correlation coefficients (ICC). Construct validity of relevant items was assessed using pairwise non-parametric correlations (Spearman’s). The SCREENS questionnaire is based on a multidimensional and formative model.

**Results:**

The SCREENS questionnaire covers six domains validated to be important factors of screen media use in children and comprises 19 questions and 92 items. Test-retest reliability (*n* = 37 parents) for continuous variables was moderate to substantial with ICC’s ranging from 0.67 to 0.90. For relevant nominal and ordinal data, kappa values were all above 0.50 with more than 80% of the values above 0.61 indicating good test-retest reliability. Internal consistency between two different time use variables (from *n* = 243) showed good correlations with rho ranging from 0.59 to 0.66. Response-time was within 15 min for all participants.

**Conclusions:**

SCREENS-Q is a comprehensive tool to assess children’s screen media habits, the screen media environment and possible related correlates. It is a feasible questionnaire with multiple validated constructs and moderate to substantial test-retest reliability of all evaluated items. The SCREENS-Q is a promising tool to investigate children screen media use.

## Background

The screen-media landscape has changed drastically during the last decade in many families with children. While the television (TV) and gaming consoles have been in the household among the majority of families for decades, the wide-scale ownership and use of new portable touchscreen-based devices as smartphones and tablets and the available applications for these devices may plausibly have changed the screen use volume and the way young people entertain themselves and communicate with friends and family members. The characteristics of the screen media environment in a typical household now often include multiple devices; TV, gaming console, smartphone, tablet, and personal computer including multiple applications with passive or interactive abilities [1]. Thus, today’s screen media are now more multifaceted and complex in nature than just a few years ago.

In recent years, researchers have become increasingly interested in investigating what determines screen media use (SMU) and the possible long-term consequences of excessive SMU. Different instruments and questionnaires have been used to assess screen time, but we are unaware of questionnaires that asses the broad screen media environment that also include use of specific media content [2], family screen media rules and other screen media habits. Most questionnaires have investigated either screen time [3–5], or media content [6–8] and the majority of the studies have addressed only TV time and computer use, and do not include screen use from other devices such as smartphones and tablets [2]. Furthermore, the target group in some of these studies have been infants or children too young to control the media use by themselves; thus measuring their exposure to screen media through their parents’ media use [2–4, 9]. Studies addressing children’s screen-time or content suggest that the content might have a greater influence on health outcomes in youth rather than the actual amount of screen-time [2, 10]. Few studies have reported test-retest reliability and validity results in screen time questionnaire instruments [11–14] but evaluations have been limited to TV- and computer time and no studies have examined the metric properties of items that attempt to capture children’s screen media use of today. One screen time questionnaire was developed with its primary focus on video- and computer gaming and showed good test-retest reliability among college students [15].

A few larger studies have used instruments which includes time spent on smartphones and tablets [16–18], one of these among children 6 months to 4 year old children [17], Nathanson [18] on 3 to 5 years old and their sleep behaviors, and the most recent study among 15 year old children [16]. However, these questionnaire instruments have not been reported systematically developed or thoroughly validated. Also, a few studies have included items to assess time spent on smartphones and tablets among toddlers or preschool children [3, 17, 18] or adolescents [16] in order to quantify time spent on screen devices. To our knowledge none of these are reported to be systematically developed or validated. Furthermore, no validated questionnaire has addressed SMU in a broader context including time spent on different devices and platforms, the context of use, the home-environment, and screen media behavior of children.

To further progress the research area of SMU and its relation to health of children and young people, a new comprehensive instrument is needed to assess children’s screen habits in addition to the broad screen media family environment that children grow up in. A new instrument will help improve the efforts to conduct more rigorous quality observational studies. The aim of this study was to develop a parent-reported standardized questionnaire to asses 6–10-year old children’s leisure time SMU and habits, that also captures the screen media environment surrounding the child. The characterization of the screen media environment also includes putative screen media specific correlates. Accurately measuring such proximal correlates in observational studies may assist in identifying possible targets for interventions that aim to change children’s screen media use. In this paper we describe the development of the SCREENS questionnaire (SCREENS-Q) including an examination of its reliability, internal consistency of independent sub-scales, and qualitative and quantitative item analyses of the final questionnaire in an independent sample of parents of children representing the target population.

## Methods

Initially a steering group was established to conduct and assess the process of developing the tool for measuring children’s SMU. Further, parents representing the target group and a panel of Danish media experts were recruited as key informants and in initial validation of the questionnaire.

### Scientific steering group

A steering group was formed to initiate and guide the development of a questionnaire to asses children’s screen media environment and its plausible proximal correlates. Members of the Steering group are the authors of this paper and are all part of the academic staff at the Center for Research in Childhood Health and Research unit for Exercise Epidemiology at the University of Southern Denmark. The steering group was responsible for initial item generation, the selection of parents and media experts, design and distribution of the questionnaire, analyses of responses from parents and experts, and drafting the final questionnaire.

### Key informants – parents

A convenience sample consisting of 10 parents of 6–8-year-old children (this age-range was chosen for convenience and because we wanted to address the youngest) were recruited for the face- and content validation interviews of the first draft of the questionnaire. Inclusion criteria for this convenience sample were being parents of children between 6 and 8-years of age (pre-school or first grade) with a regular use of screen media and we wanted parents of both boys and girls. An email invitation letter was sent to parents from public school in the local area of the city of Odense (Denmark) containing written participant information.

### Key informants – screen media experts

Ten Danish media experts were recruited to evaluate a draft of the SCREENS-Q. Criteria for being an “expert in the field of SMU” were: having published peer-reviewed scientific papers on the topic, or having authored books on media use, or being involved with descriptive national reporting of media use in Denmark. We were aware of recruiting experts of both genders, and publicly were advocates, opposed, or neutral towards children’s heavy use of screen media. The final panel of Danish media experts were representing areas of psychology, media, communication, journalism and medicine (see list of media experts in acknowledgement).

### Steps of development

**Fig. 1:**
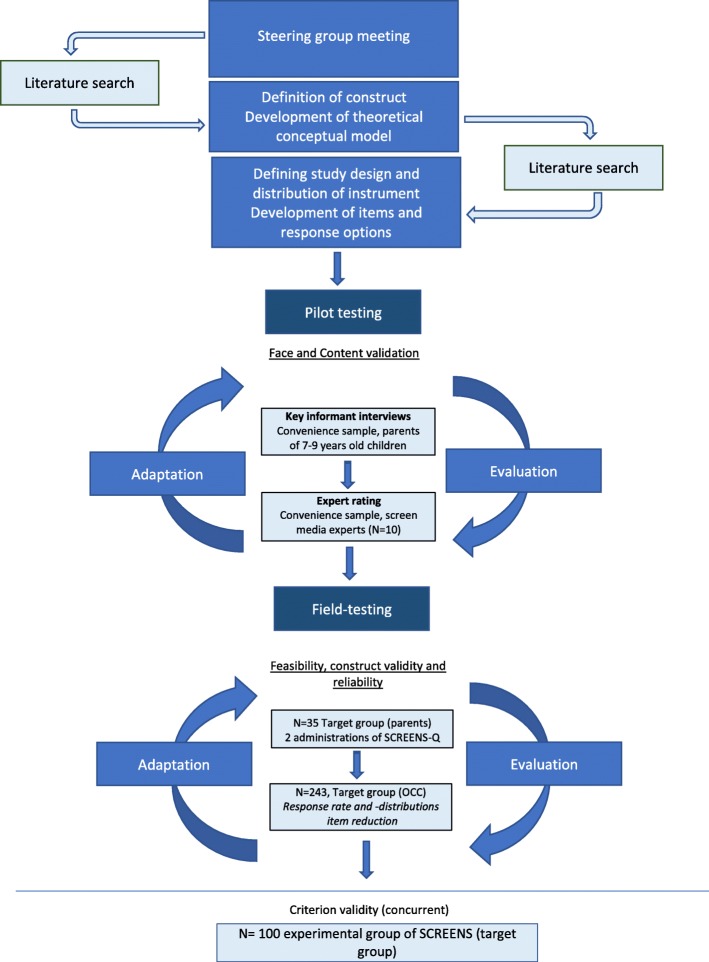
Illustration of the iterative process of the development and validation of the SCREENS-Q

The developing process of the SCREENS-Q was accomplished through an iterative process with several intertwining steps (see Fig. 1). Based on solid methodological literature [19–21], the initial process comprised the following steps: 1. Definition and elaboration of the construct to be measured, 2. Selection and formulation of items, 3. Pilot testing for face and content validity (parents and screen media experts), 4. Field testing in a sample of parents with children 7 years of age for test-retest reliability, and 5. Another field test in a larger sample of the target group from Odense Child Cohort (OCC) for assessment of construct validity and item analysis for final scale evaluation.

Steps 1, 2 and 3 were primarily qualitative evaluations. They were conducted as an iterative process in close collaboration with parents of 6–8-year-old children, the scientific steering group, and Danish screen media experts. Step 4 and 5 were primarily a quantitative evaluation of the test-retest reliability and analysis of item correlation and response distributions.

### Defining the construct and initial generation of items (steps 1 and 2)

**Fig. 2:**
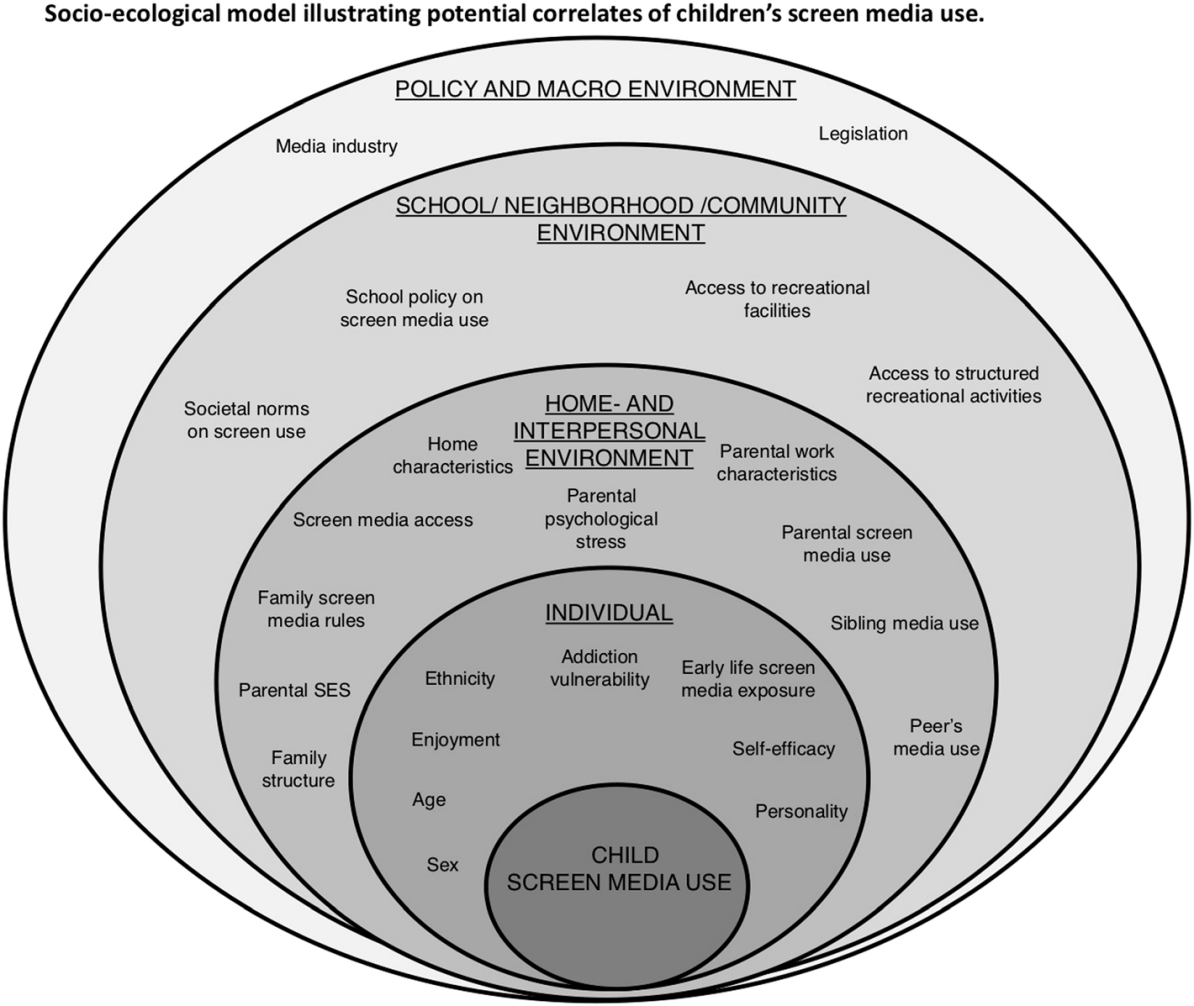
Socio-ecological model illustrating potential correlates of children’s screen media use

With the SCREENS-Q we aimed to measure children’s SMU (time and content) and specific screen-media behavior, the screen media home environment including important putative proximal correlates of children’s SMU. Several methods were used to identify relevant factors of these constructs. For the proximal correlates the scientific steering group initially established a theoretical model based on a socio-ecological model, to provide a foundation to define and comprehensively understand how various factors that may determine children’s media use are interrelated (see Fig. 2). The socio-ecological model worked as a unifying framework for identifying and investigating potential correlates of children’s SMU. Subsequently, a literature search identified and supplemented constructs from the socio-ecological model [22, 23]. Based on this model we also included relevant questionnaire items from former or ongoing studies [13, 16, 17, 24–27].

With the SCREENS-Q we aimed to assess possible direct and indirect causal factors that may influence children’s SMU. The questionnaire is multidimensional and based on a formative model [21, 28] meaning that it is intended to cover and measure all indicators that might possibly contribute to the construct “children’s SMU”. Potential causal factors may have different impacts but in a formative perspective we aimed also to identity factors with little impact. Therefore, in the initial phase we attempted to obtain a comprehensive analysis of the construct [20, 21, 28] to generate a broad list of domains and items, that were not necessarily correlated. Reduction of redundant items was carried out in later steps during pilot and field testing [20, 21, 28].

The amount of questions and items within each domain is not necessarily an expression of importance or weighing of the specific domain, but rather a question of meaningful wording and/or how accurately we wanted to measure the specific domain. This first version of SCREENS-Q was developed to use in a large ongoing birth cohort Odense Child Cohort (OCC). Therefore, relevant demographic, social and health behavior questions are obtained from measures and questionnaires in OCC (i.e. family structure, religious and ethnic origin, TV in the bedroom, other health related variables, attendance of institutions, socioeconomic status).

### Pilot testing: face and content validity (step 3)

A first draft of SCREENS-Q was developed based on the socio-ecological model, and face- and content validation was tested in a convenience sample of key informants. Ten parents of children aged 6–8 years filled out the questionnaire, while researchers were present for eventual questions about understanding and interpretation of wording of the questionnaire. Right after completing the questionnaire a semi-structured interview was conducted on relevance, importance, and if some important domains or areas of children’s SMU were missing. The key informant interviews were recorded and transcribed. Every item in the questionnaire was analyzed and revised based on the interviews, in relation to wording, understanding, interpretation, relevance and coverage for SMU in the sample of parents. Relevant changes were adapted after careful consideration in the steering group.

Fifteen Danish media experts unaffiliated with the study were contacted by telephone, informed about the project and asked if they were willing to evaluate the SCREENS-Q questionnaire. Ten of the 15 experts agreed to participate. An updated draft was sent to the ten media experts for another evaluation of face and content validity. The experts received an email with a brief description of the aim of our project, the purpose of the questionnaire, and a link to the online questionnaire in SurveyXact. They were asked not to fill it out, but to comment on every item and/or domain in the questionnaire. They were also asked to comment on wording, understanding and relevance for each item. Finally, they were asked whether the domains in the questionnaire adequately covered all significant areas of children’s use of screen media. Based on the responses and subsequent discussion in the steering group the questionnaire was further refined, and some items were modified, deleted, or added to the questionnaire.

The experience from these first steps were discussed in the scientific steering group and a final draft for field testing in the target group now comprised a questionnaire covering 6 domains, 19 questions summing up to 96 items about children’s SMU and potential correlates (see Table 1 for Domains, questions and items included in the SCREENS-Q). Step 1–3 was conducted as an iterative process from February to July 2017.

**Table 1:** Domains of screen-media use and proximal correlates included in the SCREENS-Q, example questions and response categories

Domains	Number of questions and items *(question-number in SCREENS-Q)* Chosen statistical test for reliability	Areas of interest/example questions and response category
Screen media environment	**7 questions** **43 items reduced to 39** *(3, 4, 5, 6,7 8 + 8.1, 10, 11)* Kappa, weighted kappa	Does your child have its own: laptop, PC, tablet, smartphone, TV, not-hand-held device (PlayStation/x-box/Nintendo), hand-held-device (I.e. PSP, Nintendo Switch, and Gameboy), E-reader, Other *(yes/no)?* How many of the following screen media devices are present in the household where the child lives? *(numbers)* How often has the child used the following screen media devices in the household within the past month [same devices as above]? *(5- point Likert scale; every day – never).* Access to screen medias during school time *(4- point Likert scales; never – daily)*. How often is the TV on in your home without anyone watching? *(4- point Likert scale; never – daily)* Rules for Screen media use set by the parents *(9 questions (after field-testing reduced to 5), categorial response options: agree/disagree)*
Childs Screen Media Use	**3 questions** **16 items** (9, 12, 13) ICC and BA plots	Time spent on screen medias (hours and minutes) allocated on different activities (Film/TV, games, homework, social medias, and film or musical apps) on a typical weekday/weekend day? *(none, 1–29 min, 30–59 min, 1–2 h., 2–3 h., 3-4 h, 4–5 h, > 5 h)* How many days a week does your child have screen media use the first 30 min after waking up in the morning? / the last 30 min before he/she goes to sleep *(0–5 days a week/0–2 days in the weekend*), on a typical day (weekday/weekend day) How many minutes/hours does your child use screen media before school, after school – before dinner, after dinner*? (0, 15, 30, 45, 60.90. 120, 150, 180, 240, 300, 330)*
Context of screen media use	2 questions 2 items (14, 15) Weighted kappa	When using screen media, how often does your child use more than one screen device? *(5- point Likert scale: never-always)* When your child use screen media is it then usually with: *1) you/another adult, 2) friends, 3) siblings, 4) alone*
Early exposure	1 question 4 items 2 items changed after field-testing *(17, 17.1, 17.2, 17.3)* Weighted kappa	Age when child has its own tablet/smartphone *(age 0–7)* *Instead of2 questions asking about age of daily use, we inserted 2 questions of age when child had its own PC and laptop*
Parental perception of child’s media use	1 question 16 items *(16.1–16.16)* Weighted kappa	If your child can choose activity on its own will he/she choose screen media / play outside, Screen media use helps my child; calm down, learn math, read, write, social networking, My child’s screen media use is sufficient? I am worried about my child’s SMU in relation to mental/physical health? Making rules for SMU often leads to conflicts? My child wishes to use screen medias on a daily basis. *(4-point Likert scale, totally agree-totally disagree)*
Parental Media Use	3 questions 15 items *(18, 18.1, 19)* Kappa, Weighted kappa ICC and BA plots	Parents were asked if the home was their primary place for working *(yes/no, unemployed)* and if yes, how much time they spend on work related screen time in the home *(min and hours)*. Time spent on screen medias (hours and minutes) allocated on different activities (film/TV, games, SoMe, Facetime/Skype, surfing the internet, Other:i.e. photo-, film, office programs) on a typical weekday/weekend day? *(none, 1–29 min, 30–59 min, 1–2 h, 2–3 h, 3–4 h, 4–5 h, > 5 h)*

### Field testing in the target group (step 4 and 5)

#### Step 4: examination of test-retest reliability

Another convenience sample was recruited from schools (1st grade and 2nd grade) in the municipalities of Odense and Kerteminde, Denmark. Inclusion criteria were: 1) Being parents to children at 7–9 years of age, and 2) the child must have access to- and use minimum two of the following screen media devices in the household: Tablet, smartphone, TV, gaming console or computer. In total 35 parents agreed to participate in this field testing for test-retest reliability. The questionnaire was sent to the parents, and responses collected electronically. The participants were asked to fill out the SCREENS-Q twice, separated by a two-week interval. Step 4 was conducted in November 2017 and December 2017.

#### Step 5: construct validity and item analysis

After evaluating test-retest reliability in the convenience sample, the SCREENS-Q was implemented in OCC, an ongoing closed birth cohort initiated in 2010–2012 [24]. The evaluation of construct validity was done with two items measuring screen time (item 9 and 13 from *n* = 243). Furthermore, item analysis (based on descriptive analysis of data and qualitative evaluation of response patterns and feedback), and feasibility (willingness and time to fill out the SCREENS-Q) was evaluated on a subsample of parents from the cohort (n = 243). Items would be deemed redundant if they had too little variation. Item responses were analyzed to investigate whether any answer categories were unexpectedly unused/not answered and therefore seemed redundant or irrelevant. Participating parents were asked to fill out the SCREENS-Q on a tablet while attending the 7-year old examination at the hospital. If the child did not attend the planned 7-year old examination the questionnaire was sent to the parents by email. Step 5 was conducted on data from ultimo November 2017 to primo March 2018.

### Data management

The questionnaire was distributed and answered online. In the pilot testing (step 3) SurveyXact was used for management and initial response analysis. For the field testing, a professional data entry organization (Open Patient data Explorative Network) entered the data in the survey/data management software REDCap. A series of range and logical checks were undertaken to clean the data.

### Statistical methods

To determine test–retest reliability selected relevant items were compared between the first and second administrations of SCREENS-Q during field testing (*n* = 35). For categorical/binominal variables (questions 4, 5 and 11) levels of agreement were determined using Kappa coefficients which were defined as poor/slight (κ = 0.00–0.20), fair (κ = 0.21–0.40), moderate (κ = 0.41–0.60), substantial (κ = 0.61–0.80) and almost perfect (κ = 0.81–1.00) [29]. Reliability for questions on an ordinal scale (item 3, 6, 17 and 11) was assessed using weighted kappa and/or intra-class correlation (ICC) as these estimates are identical if the weights in kappa are quadratic [28]*.* To avoid excluding items with a low Kappa value despite showing a high percent agreement (due to a high percent of responses in one category, creating instability in the Kappa statistic) it was decided that items with a κ > 0.60 and/or percent agreement ≥60% were considered to have acceptable reliability [30, 31].

Test-retest reliability of continuous variables (item 9, 13 and 19) was evaluated by calculating ICC and standard error of measurement. An ICC value of 0.75 or higher was considered to represent a good level of agreement. ICC values of 0.50–0.74 were considered to represent moderate reliability and those below 0.50 represented poor reliability. Bland-Altman plots were created, and 95% limits of agreement calculated to investigate agreement between the first and second administration of the SCREENS-Q for continuous variables.

As SCREENS-Q is a multidimensional tool based on a formative model, item analyses were primarily done by qualitative evaluation of distributions and usefulness. Factor analysis and definition of internal consistency does not apply, as items are not assumed to be internally correlated in a formative model [21]. This applies to all items in the questionnaire except from questions 9 and 13 that each can be summarized to provide a total screen time use variable. Construct validity is about consistency – not accuracy [19, 28]. Thus construct validity of these questions was assessed using pairwise non-parametric correlations (Spearman’s) and 95% CI calculated by bootstrap estimations with 1000 reps [32].

All analyses were conducted in Stata/IC 15.

## Results

Following the five iterative steps of developing, field testing and evaluating validity and reliability resulted in a final version of SCREENS-Q comprising six domains, nineteen questions and 92 items representing factors from individual, social (interpersonal) and physical (home) environment of our socio-ecological model. (see Table 1 and Additional file 1 (Danish) and Additional file 2 (English) for the full version).

### Validity

Our two groups of key informants 1) ten parents (aged 30–49, 70% mothers) of ten children 6–8 years of age (6 boys and 4 girls) and 2) ten Danish media-experts (6 females, 4 males) from very different areas of education, including the scientific fields of psychology, media, communication, journalism and medicine, evaluated face and content validity. Wording, understanding, interpretation and coverage was confirmed by both groups. Parents suggested that one originally drafted item about *“whether the child is using the media on and off, and thereby having many, but short, bouts of SMU”,* was not an issue for children 6–8 years of age, so that question was omitted. They also suggested that SMU during school hours (educational wise) might influence leisure time SMU. Based on the key informant interviews additional items were added (questions 6, 7, 8 and 8.1). The media experts’ biggest concern was that it could be difficult to capture children’s SMU and behavior or its determinants in a questionnaire because of the complexity of screen media behavior. Some experts emphasized that we should be aware of also aiming to capture the positive effects of SMU. Thus, question 16 was expanded with several items addressing possible positive effects of SMU (items 16.3, 16.4, 16.5, 16.6, 16.7, 16.9, 16.10, 16.11). Other experts emphasized the importance of asking about relations and context (i.e.: who is the child with during SMU, rules for SMU etc.). Therefore, question 11, 14 and 15 were refined and extended. The final version of SCREENS-Q contains six domains validated to be important factors of defining the construct of “children’s SMU and behavior”. Five of the six domains address the child’s SMU, screen media preferences (device, content), screen media behavior (when and with whom and on what device and platform) and the screen home environment and comprises 16 questions and 77 items, and one domain addresses the parents SMU in the home (two questions and 15 items) (see Additional file 1 for the full SCREENS-Q).

Construct validity was assessed by investigating internal consistency between two different questions asking about children’s SMU (time in hours and minutes) on a typical weekday and a weekend day (question 9 and question 13) in the second field test with *n* = 243 parents.

Spearman’s rho showed good correlation (rho ranging from 0.59 to 0.66) between the two different ways of measuring time spend on screen medias (Table 2).

**Table 2:** Construct validity (question 9 measured against question13 (*n* = 243))

Construct validity Comparing time (minutes) obtained by question 9 to time obtained by question 13 (at the same timepoint)	Spearman’s correlation: Rho (95%CI)
On a typical weekday	0.63 (0.54–0.72)
On a typical weekend-day	0.59 (0.49–0.69)
Time summed for a whole week^a^	0.66 (0.57–0.75)
Mean time/day^b^	0.66 (0.57–0.75)

*p*-value for all correlations < 0.0001

^a^ (minutes on a typical weekday × 5) + (minutes on a typical weekend day × 2)

^b^ ((minutes on a typical weekday × 5) + (minutes on a typical weekend day × 2)/7)

### Reliability

Test-retest reliability was investigated in the convenience sample of thirty-five parents. Thirty-five completed Q1 and of these *n* = 31 responded to Q2, which gave us n = 31 eligible for the test-retest reliability analysis. Of the 31 parents (25 mothers and 6 fathers of 19 boys and 12 girls) who filled out SCREENS-Q twice, *n* = 11 completed the questionnaire within the expected 2–2½ weeks, n = 11 within 3–3½ weeks and *n* = 9 after 4–4½ week. Mean time to follow up was 22.5 (SD 6.5) days.

For continuous variables (questions and items 9,13,18.1 and 19) ICC and BA plots were calculated and test-retest reliability was considered moderate to good as ICC for all examined items were between 0.67 to 0.90 (see Table 3, where also Standard Errors of Mean, Mean differences and Limits of agreement are presented). For all other items, kappa or weighted kappa, and observed agreement were calculated if applicable and showed high values for reliability. All kappa values for items included in the present version of the questionnaire were all above 0.50 and 80% of the kappa values were above 0.61 indicating good test-retest reliability, ranging from moderate to substantial. Less than 10% of the items/questions returned low kappa values despite high observed agreement due to too high response in one category. None of the observed agreement values were below 60% and the majority (65%) showed an observed agreement value ≥90% (see Table 4 for an overview).

**Table 3:** Test-retest reliability of child- and parent screen time use (*N* = 31)

Question (measure) assessed (all units of measurement are minutes)	Statistical assessment of test-retest reliability
Question 9	
Time spent using screen media on a typical weekday, (child)	ICC = 0.72 (95%CI: 0.52–0.86) SEM = 38.84 min/day (95% CI: 30.09–50.13) Mean difference = 2.74 Limits of agreement = (− 105.02, 110.51)
Time spent using screen on a typical weekend-day, (child)	ICC = 0.67 (95%CI: 0.47–0.84) SEM = 59.88 min/day (95% CI: 46.75–76.70) Mean difference = 18.23 Limits of agreement = (− 146.93, 183.39)
Mean min per day, child (min week-day × 5) + (min per weekend day × 2)/7(child)	ICC = 0.75 (95% CI 0.58–0.88) SEM = 38.52 min/day (95% CI 29.94–49.56) Mean difference = 7.17 min/day Limits of agreement = (− 99.67, 114.00)
Question 13	
Time spent using screen media on a typical weekday, (child)	ICC = 0.81 (95%CI: 0 .67–0.90) SEM = 26.66 min/day (95% CI: 20.81–34.14) Mean difference = 2.42 Limits of agreement = (− 72.76, 77.60)
Time spent using screen media on a typical weekend-day, (child)	ICC = 0.90 (95%CI: 0.81–0.95) SEM = 47.29 min/day (95% CI: 36.91- = 60.59) Mean difference = 18.87 Limits of agreement = (− 109.27, 147.01)
Mean min per day, child (min week-day × 5) + (min per weekend day × 2)/7(child)	ICC 0.88 (95%CI: 0.79–0.94) SEM = 27.05 min/day (95% CI: 21.11–34.65) Mean difference = 7.12 Limits of agreement = (− 67.93, 82.16)
question 19	
Time spent using screen media on a typical weekday, (parent)	ICC = 0.68 (95%CI: 0.48–0.84) SEM = 60.47 min/day (95% CI: 47.33–77.26) Mean difference = 10.16 Limits of agreement = (− 160.49, 180.81)
Time spent using screen media on a typical weekend-day, (parent)	ICC = 0.80 (95%CI: 0.66–0.90) SEM = 63.01 min/day (95% CI: 49.22–80.66) Mean difference = 2.42 Limits of agreement = (− 175.78, 180.62)
Mean min per day, parent (min week-day × 5) + (min per weekend day × 2)/7(parent)	ICC = 0.75 (95%CI: 0.59–0.88) SEM = 54.54 min/day (95% CI: 42.65–69.76) Mean difference = 7.95 Limits of agreement = (− 145.85, 161.75)
question 18	
Work-related time spent on screen media on weekdays in the home, (parent)	ICC = 0.73 (95%CI: 0.55–0.87.) SEM = 63.86 min/day (95% CI: 49.70–82.04) Mean difference = − 3.87 Limits of agreement = (− 182.86, 175.12)
Work-related time spent on screen media on weekend-days in the home, (parent)	ICC = 0.69 (95%CI: 0.50–0.84) SEM = 26.57 min/day (95% CI: 20.76–34.02) Mean difference = − 5.81 Limits of agreement = (− 80.06, 68.44)

*ICC* Intraclass Correlation Coefficient, *SEM* Standard Error of Mean, *LOA* Limits of Agreement, *CI* confidence interval

**Table 4:** Summary of reliability assessment by domains

Domain	Test-retest reliability^a^ (*N* = 31)
Screen Media Environment (7 questions- 39 items)	**Substantial to almost perfect** (kappa values 0.76 to 0.93) **Observed agreement** range from **61 to 100%** (only 5% had less than 80% of observed agreement)
Childs Screen Media Use (3 questions-16 items)	**Moderate to good** (ICC = 0.67 to 0.90) **Substantial** (kappa values 0.68 to 0.79) **Observed Agreement** range from **89.5 to 95.2%**
Context of screen media use (2 questions- 2 items)	**Moderate to Substantial** (kappa values: 0,41 and 0.72) **Observed agreement**: 88.5 and 96.1%
Early exposure (1 question – 4 items)	**Almost perfect** (kappa values 0.89 to 0.97) **Observed agreement: 98.4–99.6%**
Parental perception of child’s media use behavior (1 question- 16 items)	**Fair to almost perfect** (kappa values 0.37 to 0.85) **Observed agreement** range from **81.5 to 96.4%**
Parental Media Use (3 questions-15 items)	**Moderate to good** (ICC = 0.69 to 0.75)

^a^For binary categorical response options ordinary kappa was calculated, for ordinal response options weighted kappa was calculated and for continuous variables intraclass correlation coefficients (ICC) and Limits of agreement (LOA) were calculated

### Item analysis

Data for item analysis is from *n* = 243 parents (*n* = 60 fathers, *n* = 182 mothers, n = 1 “relation to the child” not stated) of n = 243 children (*n* = 142 boys, *n* = 101 girls) participating in the OCC. The majority (48%) of the parents had a first stage tertiary education (short or long college, i.e. bachelor’s degree), 26 percentage had completed higher education at university (i.e. master’s degree or above) and 26 percentage of the parents had completed upper secondary school or a vocational education.

Response rate and completeness was high (98.4%) and quantitative and qualitative item analysis did not suggest further deletion or modification of items. However, the parents of the cohort (n = 243) primarily filled out the questionnaire during the time, the child underwent the biennial examination in the cohort, which gave them the opportunity to ask for further explanation when answering. A few parents felt that the question about age of first regular daily SMU was hard to answer and did not make as much sense for them as “age when the child had its own device”. These questions about first regular daily SMU showed good, but slightly lower kappa values (0.60 and 0.81, respectively) than questions reporting age when the child owned its first personal device (smartphone or tablet) (kappa value 0.89 and 0.97). It seemed harder for parents to estimate age of first regular daily screen media as disagreement between first and second response could differ 2 years. Therefore, in the final version of SCREENS-Q, questions about age of first regularly daily are replaced with two more questions about age, when the child had its own device (personal computer and/or laptop).

In question 4 we asked: *“Thinking about the last month which of the following devices have your child used?*” Respondents had two possible response categories: “yes/no” for each of the displayed screen devices, which gave very limited information about the child’s actual use. Based on the modest variation in response in the sample we decided to modify and expand the question to *“How often has the child used the following screen media devices in the household within the past month?”* and include five response categories (*1. “Every day or almost every day of the week”, 2. “4-5 days per week”, 3. “2-3 days per week”, 4. “1 day or less per week”, and 5. “Never*”).

For question 11 about rules for media use we initially had nine statements about rules with different formulations like *“the child can decide on its own how much time it spends on screen media”* and another phrased like *“we have firm rules for how much time the child can spend on screen media”*. We tested the overall agreement of these somewhat similar questions to whether parents responded in a similar way to these phrasing. Overall agreement was high (from 67.74 to 90.32%) and we decided that the final version should include only five of them. The reliability of test-retest of all items about rules (question 11) was moderate to substantial with kappa values from 0.71–0.79 (For a single item (11.b) only fair *k* 0.30) (see Table 4 for a summary of reliability measures by domains).

### Feasibility

Feasibility in the field test sample was considered good as all parents (*n* = 243) present for the child’s 7-year-old examination, and *n* = 239 (98.4%) completed the questionnaire without any missing answers. All completed questionnaires were completed within 15 min if the completion was not interrupted by other tasks.

## Discussion

The main focus of this paper was to describe the development of SCREENS-Q designed to assess 6–10-year old children’s leisure time screen media use and behavior, the screen media environment, and its proximal correlates, and to determine multiple domains of its validity and test-retest reliability. It was developed based on a conceptual model informed by literature and face and content validity were established by involving screen media experts and end users (parents) of the SCREENS-Q in an iterative process. Internal consistency was assessed by field test in a larger sample and estimated high for screen media time use and test-retest reliability was moderate to substantial for all items. Overall, the SCREENS-Q provides an up-to-date standardized questionnaire for parent reported assessment of children’s leisure time SMU and habits and possible screen media specific correlates.

To our knowledge SCREENS-Q is the first questionnaire battery to comprehensively assess children’s screen media habits, the screen media home environment, potential determinants of habits that may assist in identifying possible targets for intervention. These include possible individual level factors, home- and interpersonal environmental level factors, and a few school/neighborhood/community level factors according to our suggested socio-ecological model of child screen media habits.

Test-retest reliability of the SCREENS-Q was moderate to substantial (ICC ranging from 0.67 to 0.90) for all items which is similar but higher than the “acceptable or better” test-retest reliability of screen media questions in the HAPPY study by Hinkley et al. (ICC ranging from 0.31 to 0.84) [27]. This difference might be due to more detailed and accurate answer categories in the SCREENS-Q (hours and minutes within 15 min for each screen media device and activity) compared to the HAPPY study where parents of preschoolers were asked to estimate time in hours that their child engaged in screen behaviors on a typical weekday and weekend day.

There are limitations to this study that need to be addressed and considered when interpreting the results and application of the SCREENS-Q. The SCREENS-Q was developed as a parent reported questionnaire for children aged 6–10 years of age. Proxy-reporting by parents can have mixed validity; for example parent reporting of children’s pain [33] has been reported to have low agreement, while parent reporting of health related quality of life in children has shown to be valid and reliable [34]. Questions assessing time spent participating in specific behaviors may be particularly difficult to accurately report [35]. Parents’ awareness of and ability to accurately recall the time their child spends in a specific behavior might be limited and thus the answers prone to lower validity and reliability than objectively measured behavior. In addition, parent reporting may be prone to underestimation due to social desirability response bias as many parents today may consider children’s high SMU outside the social norm [36].

Another limitation is the relatively small non-population-based sample of parents for test-retest reliability. Most reliability measures are sample dependent, and despite internal consistency assessment and reliability measures showed moderate to high agreement and reliability for all items, these estimates may not reflect the general target population [21]. The average time between the administrations of the two questionnaires were 22.5 days and the difference between the responses may also represent true differences.

This first version of SCREENS-Q was developed in corporation with Danish parents and media experts to especially capture the SMU of 6–10-year-old Danish children and only tested on 7–8-year-old children in this study. In accordance with suggested age-limits of self-report in children [37, 38] we believe that from the age of approximately 11 years children will be able to self-report their screen use and behavior with higher accuracy compared with parental report. Therefore, a self-report version of the SCREENS-Q for older children and young people will need to be developed and evaluated. We collected data on parental screen media use, as we hypothesized that parents screen media use might be a determinant of their child’s screen media use. For pragmatic reasons we only asked the attending parent about screen media use, which might be a limitation as mothers and fathers screen media use could relate differently to the child’s media use. To fully address parental screen media use as a possible determinant in a future study it is possible to administer the two questions to each parent.

The generalizability might be restricted to young Danish children and future investigations of validity and reliability in other samples, nationalities and cultures are warranted. School policy on SMU during school time might also have an impact on children’s leisure time SMU. That domain is not well covered in the SCREENS-Q. Furthermore, although the questionnaire included numerous items it was completed quickly by all respondents if completed uninterrupted. Yet, in the population-based field test sample, the majority of parents, were well educated. Thus, it remains unclear if less educated parents would have similar answers, completeness and response times. Finally, although we conducted a comprehensive analysis of the construct to generate a broad list of domains and items, the questionnaire may still lack coverage of some elements of the possible proximal correlates of children’s screen media use.

The strength of this study is the careful conceptual development, involving experts and end users, and that the questionnaire concurrently covers wide domains of screen media behavior and factors that might influence children’s SMU.

## Conclusion

The SCREENS-Q was developed to meet the research needs of a comprehensive tool to assess screen habits of children and the possible screen media related correlates based on a socio-ecological perspective. We have developed a feasible questionnaire and validated multiple constructs and found moderate to substantial test-retest reliability of all inspected items. Conclusively, the SCREENS-Q is a promising tool to investigate children’s SMU. We are planning a future study to carefully examine the criterion validity of the time use items, and we are currently collecting SCREENS-Q data in a population-based sample to examine the relationship of the proximal correlates of screen media habits with SMU in children.

## Supplementary information

**Additional file 1.** The final version of the SCREENS-Q in Danish (original)

**Additional file 2.** Translated version of the SCREENS-Q in English

## Data Availability

The datasets used and/or analyzed during the current study are available from the corresponding author on reasonable request.

## Abbreviations

CI: Confidence interval
ICC: Intraclass correlation coefficient
OCC: Odense Child Cohort
SCREENS-Q: SCREENS questionnaire
SEM: Standard Error of Mean
SMU: Screen media use
TV: Television
