# Supplementary material for: The development of a questionnaire to assess leisure time screen-based media use and its proximal correlates in children (SCREENS-Q)
Source: BMC Public Health. 2020 May 12;20:664. doi: 10.1186/s12889-020-08810-6 (PMC7216486; doi:10.1186/s12889-020-08810-6)
Supplement: Supplementary file 1 — Additional file 1. The final version of the SCREENS-Q in Danish (original) [file 12889_2020_8810_MOESM1_ESM.docx]

| SCREENS spørgeskemaet har til formål at undersøge børns skærmmedie forbrug- og adfærd.  Vi håber, at du vil besvare spørgeskemaet så detaljeret som muligt, på vegne af barnet. Bor barnet kun noget af tiden hos dig, tag da udgangspunkt i de skærmaktiviteter der foregår, når barnet er hos dig.  **Spørgeskemaet tager ca. 15 minutter at udfylde.** |
| --- |

## 1. Hvad er din relation til barnet?

🞏 Far

🞏 Stedfar

🞏 Mor

🞏 Stedmor

🞏 Andet

Hvis andet, skriv hvad:

________________________________________

**2. Hvor mange personer bor der i husstanden?**
Medregn også personer, som kun delvist har bopæl i husstanden, eksempelvis delebørn.

_______

| Barnets adgang til skærmmedier. |
| --- |

**3. Hvor mange af følgende skærmenheder er der i den husstand, hvor barnet bor?**
Medregn alle skærmenheder - også enheder der tilhører personer, der kun delvist har fast bopæl i husstanden, og enheder, som barnet ikke har adgang til. Enheder der aldrig bruges skal ikke medregnes.

|  | Angiv ét svar for hver skærmenhed | | | | | |
| --- | --- | --- | --- | --- | --- | --- |
|  | 0 | 1 | 2 | 3 | 4 | 5 eller flere |
| Bærbar computer | 🞏 | 🞏 | 🞏 | 🞏 | 🞏 | 🞏 |
| Stationær computer | 🞏 | 🞏 | 🞏 | 🞏 | 🞏 | 🞏 |
| Tablet/iPad | 🞏 | 🞏 | 🞏 | 🞏 | 🞏 | 🞏 |
| Smartphone | 🞏 | 🞏 | 🞏 | 🞏 | 🞏 | 🞏 |
| TV | 🞏 | 🞏 | 🞏 | 🞏 | 🞏 | 🞏 |
| Ikke-håndholdt spillekonsol (F.eks. Xbox, Playstation, Nintendo) | 🞏 | 🞏 | 🞏 | 🞏 | 🞏 | 🞏 |
| Håndholdt spillekonsol (F.eks. PSVita, PSP, Nintendo Switch, Gameboy) | 🞏 | 🞏 | 🞏 | 🞏 | 🞏 | 🞏 |
| E-reader (E-bogslæser) | 🞏 | 🞏 | 🞏 | 🞏 | 🞏 | 🞏 |
| Andet | 🞏 | 🞏 | 🞏 | 🞏 | 🞏 | 🞏 |

Hvis andet, skriv hvad:

________________________________________

**4. Hvor ofte har barnet brugt følgende skærmenheder i husstanden inden for den seneste måned?**
Medregn kun skærmenheder, som tilhører husstanden.

|  | *Sæt ét kryds per skærmenhed* | | | | |
| --- | --- | --- | --- | --- | --- |
|  | Hver dag eller næsten hver dag i ugen | 4-5 dage om ugen | 2-3 dage om ugen | 1 dag eller sjældnere per uge | Aldrig |
| Bærbar computer | 🞏 | 🞏 | 🞏 | 🞏 | 🞏 |
| Stationær computer | 🞏 | 🞏 | 🞏 | 🞏 | 🞏 |
| Tablet/iPad | 🞏 | 🞏 | 🞏 | 🞏 | 🞏 |
| Smartphone | 🞏 | 🞏 | 🞏 | 🞏 | 🞏 |
| TV | 🞏 | 🞏 | 🞏 | 🞏 | 🞏 |
| Ikke-håndholdt spillekonsol (F.eks. Xbox, Playstation, Nintendo) | 🞏 | 🞏 | 🞏 | 🞏 | 🞏 |
| Håndholdt spillekonsol (F.eks. PSVita, PSP, Nintendo Switch, Gameboy) | 🞏 | 🞏 | 🞏 | 🞏 | 🞏 |
| E-reader (E-bogslæser) | 🞏 | 🞏 | 🞏 | 🞏 | 🞏 |
| Andet | 🞏 | 🞏 | 🞏 | 🞏 | 🞏 |

Hvis andet, skriv hvad:

________________________________________

## 5. Angiv om barnet har sin egen af de følgende skærmenheder.

|  | *Sæt ét kryds per skærmenhed* | |
| --- | --- | --- |
|  | Ja | Nej |
| Bærbar computer | 🞏 | 🞏 |
| Stationær computer | 🞏 | 🞏 |
| Tablet/iPad | 🞏 | 🞏 |
| Smartphone | 🞏 | 🞏 |
| TV | 🞏 | 🞏 |
| Ikke-håndholdt spillekonsol (F.eks. Xbox, Playstation, Nintendo) | 🞏 | 🞏 |
| Håndholdt spillekonsol (F.eks. PSVita, PSP, Nintendo Switch, Gameboy) | 🞏 | 🞏 |
| E-reader (E-bogslæser) | 🞏 | 🞏 |
| Andet | 🞏 | 🞏 |

Hvis andet, skriv hvad:

________________________________________

## 6. Medbringer barnet nedenstående skærmenheder i skolen?

| Sæt ét kryds i hver linje | | | |  |
| --- | --- | --- | --- | --- |
| Ja, dagligt eller næsten dagligt | Ja, ugentligt | Ja, sjældnere end ugentligt | Nej, aldrig |  |
| Smartphone | 🞏 | 🞏 | 🞏 | 🞏 |
| Tablet | 🞏 | 🞏 | 🞏 | 🞏 |
| Bærbar computer | 🞏 | 🞏 | 🞏 | 🞏 |
| Håndholdt spillekonsol (F.eks. PSVita, PSP, Nintendo Switch, Gameboy) | 🞏 | 🞏 | 🞏 | 🞏 |

## 7. Bruger barnet tablet, smartphone eller computer i forbindelse med undervisningen i skolen?

🞏 Ja, dagligt

🞏 Ja, ugentligt

🞏 Ja, sjældnere end ugentligt

🞏 Nej, aldrig

🞏 Ved ikke

## 8. Bruger barnet tablet, smartphone eller anden skærmenhed i frikvarterne - eksempelvis til at spille på?

**Se bort fra brug af smartphone til at komme i kontakt med forældre**

🞏 Ja, dagligt

🞏 Ja, ugentligt

🞏 Ja, sjældnere end ugentligt

🞏 Nej, aldrig

🞏 Ved ikke

## 8.1 Bruger barnet tablet, smartphone eller anden skærmenhed i SFO-tiden - eksempelvis til at spille på?

**Se bort fra brug af smartphone til udelukkende at komme i kontakt med forældre**

🞏 Ja, dagligt

🞏 Ja, ugentligt

🞏 Ja, sjældnere end ugentligt

🞏 Nej, aldrig

🞏 Ved ikke

🞏 Mit barn går ikke i SFO

| **Barnets forbrug og indhold på skærmmedier.** |
| --- |

**9. Inden for den seneste måned, hvor meget tid har barnet typisk brugt per dag på følgende skærmaktiviteter i fritiden?**
Giv ét svar i hver linje - både for en typisk hverdag og weekenddag (min = minutter, t = timer)

|  | Hverdag (tid per dag) | | | | | | | | Weekenddag (tid per dag) | | | | | | | |
| --- | --- | --- | --- | --- | --- | --- | --- | --- | --- | --- | --- | --- | --- | --- | --- | --- |
|  | Ingen | 1-29 min | 30-59 min | 1-2 t | 2-3 t | 3-4 t | 4-5 t | 5 t el. mere | Ingen | 1-29 min | 30-59 min | 1-2 t | 2-3 t | 3-4 t | 4-5 t | 5 t el. mere |
| Film, TV-serier, Youtube videoklip/film, underholdningsprogrammer | 🞏 | 🞏 | 🞏 | 🞏 | 🞏 | 🞏 | 🞏 | 🞏 | 🞏 | 🞏 | 🞏 | 🞏 | 🞏 | 🞏 | 🞏 | 🞏 |
| Spil (på smartphone, tablet, spilkonsol, PC) | 🞏 | 🞏 | 🞏 | 🞏 | 🞏 | 🞏 | 🞏 | 🞏 | 🞏 | 🞏 | 🞏 | 🞏 | 🞏 | 🞏 | 🞏 | 🞏 |
| Skolerelaterede opgaver med brug af skærmmedier | 🞏 | 🞏 | 🞏 | 🞏 | 🞏 | 🞏 | 🞏 | 🞏 | 🞏 | 🞏 | 🞏 | 🞏 | 🞏 | 🞏 | 🞏 | 🞏 |
| Videosamtale (f.eks. Facetime, Skype) | 🞏 | 🞏 | 🞏 | 🞏 | 🞏 | 🞏 | 🞏 | 🞏 | 🞏 | 🞏 | 🞏 | 🞏 | 🞏 | 🞏 | 🞏 | 🞏 |
| Sociale medier eller andre kommunikationstyper (Facebook, Messenger, Twitter, WhatsApp, Snapchat, Instagram, E-mail, SMS) | 🞏 | 🞏 | 🞏 | 🞏 | 🞏 | 🞏 | 🞏 | 🞏 | 🞏 | 🞏 | 🞏 | 🞏 | 🞏 | 🞏 | 🞏 | 🞏 |
| Andet (F.eks. tegneprogrammer, lave musical- eller stopmotion film) | 🞏 | 🞏 | 🞏 | 🞏 | 🞏 | 🞏 | 🞏 | 🞏 | 🞏 | 🞏 | 🞏 | 🞏 | 🞏 | 🞏 | 🞏 | 🞏 |

Hvis du har svaret **andet**, giv eksempler på hvad: ________________________________________

| **Medieadfærd i hjemmet** |
| --- |

## 10. Sker det at fjernsynet er tændt, mens I ikke ser på det?

🞏 Ja, dagligt eller næsten dagligt

🞏 Ja, ugentligt

🞏 Ja, sjældnere end ugentligt

🞏 Nej, aldrig

🞏 Har ikke fjernsyn i husstanden

| **Eventuelle regler for barnets brug af skærmmedier.** |
| --- |

## 11. Angiv om du er enig eller uenig i nedenstående udsagn omkring barnets skærmforbrug derhjemme:

|  | Enig | Uenig |
| --- | --- | --- |
| a. Barnet skal altid spørge om lov, før han/hun bruger skærmmedier | 🞏 | 🞏 |
| b. Der er faste rammer for, hvor meget tid barnet må bruge skærmmedier | 🞏 | 🞏 |
| c. Der er faste rammer for, hvornår på dagen barnet må bruge skærmmedier | 🞏 | 🞏 |
| d. Der er faste rammer for, hvad barnet må spille | 🞏 | 🞏 |
| e. Der er faste rammer for, hvilke film, Youtube klip, tv-serier og underholdningsprogrammer barnet må se | 🞏 | 🞏 |

| **Hvornår på dagen bruger barnet skærmmedier.** |
| --- |

**12. Hvor mange dage på en typisk uge bruger barnet skærmmedier i de følgende tidsrum?**
(F.eks.: Ser TV, spiller spil, laver skolerelaterede ting med skærmmedier)
Giv ét svar i hver linje - både for hverdage og weekenddage

|  | Antal hverdage per uge | | | | | | Antal weekenddage per uge | | |
| --- | --- | --- | --- | --- | --- | --- | --- | --- | --- |
|  | 0 dage | 1 dag | 2 dage | 3 dage | 4 dage | 5 dage | 0 dage | 1 dag | 2 dage |
| ..inden for en halv time efter at han/hun vågner om morgenen? | 🞏 | 🞏 | 🞏 | 🞏 | 🞏 | 🞏 | 🞏 | 🞏 | 🞏 |
| ..inden for en halv time før han/hun skal lægge sig til at sove om aftenen? | 🞏 | 🞏 | 🞏 | 🞏 | 🞏 | 🞏 | 🞏 | 🞏 | 🞏 |

13. Hvor meget tid bruger barnet på skærmmedier på en typisk dag i de følgende tidsrum? **Angiv antal timer og minutter per dag i hver linje (min = minutter, t = timer):**

|  | Ingen | 1-15 min | 15-30 min | 30- 45 min | 45-60 min | 1-11/2 t | 11/2-2 t | 2 -21/2t | 21/2-3 t | 3-4 t | 4-5 t | Mere end 5 t |
| --- | --- | --- | --- | --- | --- | --- | --- | --- | --- | --- | --- | --- |
| Hverdag (Før skole) | 🞏 | 🞏 | 🞏 | 🞏 | 🞏 | 🞏 | 🞏 | 🞏 | 🞏 | 🞏 | 🞏 | 🞏 |
| Hverdag (Efter skole men før aftensmad) | 🞏 | 🞏 | 🞏 | 🞏 | 🞏 | 🞏 | 🞏 | 🞏 | 🞏 | 🞏 | 🞏 | 🞏 |
| Hverdag (Efter aftensmad) | 🞏 | 🞏 | 🞏 | 🞏 | 🞏 | 🞏 | 🞏 | 🞏 | 🞏 | 🞏 | 🞏 | 🞏 |
| Weekenddag (Før kl. 12) | 🞏 | 🞏 | 🞏 | 🞏 | 🞏 | 🞏 | 🞏 | 🞏 | 🞏 | 🞏 | 🞏 | 🞏 |
| Weekenddag (Efter kl. 12 men før aftensmad) | 🞏 | 🞏 | 🞏 | 🞏 | 🞏 | 🞏 | 🞏 | 🞏 | 🞏 | 🞏 | 🞏 | 🞏 |
| Weekenddag (Efter aftensmad) | 🞏 | 🞏 | 🞏 | 🞏 | 🞏 | 🞏 | 🞏 | 🞏 | 🞏 | 🞏 | 🞏 | 🞏 |

| **Hvordan bruger barnet skærmmedier.** |
| --- |

**14. Når barnet bruger skærmmedier, hvor ofte bruger han/hun da mere end én skærmenhed ad gangen?**
(F.eks. ser TV og bruger tablet samtidig)

🞏 Aldrig

🞏 Sjældent

🞏 Ind i mellem

🞏 Ofte

🞏 Altid

**15. Når barnet bruger skærmmedier, er det så oftest...**
Sæt kun ét kryds

🞏 ..sammen med dig/andre voksne?

🞏 ..sammen med venner?

🞏 ..sammen med søskende?

🞏 ..alene?

16. Herunder ses en liste med udsagn, som handler om barnets brug af skærmmedier. **Du bedes angive hvor enig du er i hvert af udsagnene.**

|  | Helt enig | Delvist enig | Delvist uenig | Helt uenig |
| --- | --- | --- | --- | --- |
| Hvis barnet har frit valg, så vil han/hun næsten altid vælge at bruge sin tid på skærmaktiviteter | 🞏 | 🞏 | 🞏 | 🞏 |
| Hvis barnet har frit valg, så vil han/hun næsten altid vælge at bruge sin tid på leg uden skærm | 🞏 | 🞏 | 🞏 | 🞏 |
| Brug af skærmmedier i fritiden styrker barnets fællesskab med andre børn | 🞏 | 🞏 | 🞏 | 🞏 |
| Brug af skærmmedier hjælper ofte barnet med at falde til ro | 🞏 | 🞏 | 🞏 | 🞏 |
| Barnet og jeg bruger ofte skærmmedier sammen | 🞏 | 🞏 | 🞏 | 🞏 |
| Barnets brug af skærmmedier lægger op til mange hyggelige snakke med mit barn | 🞏 | 🞏 | 🞏 | 🞏 |
| Barnets brug af skærmmedier i fritiden styrker hans/hendes kreativitet og fantasi | 🞏 | 🞏 | 🞏 | 🞏 |
| Barnet har en passende mængde af skærmtid i fritiden | 🞏 | 🞏 | 🞏 | 🞏 |
| Brug af skærmmedier i fritiden er en hjælp for barnet i forhold til at lære at skrive og stave | 🞏 | 🞏 | 🞏 | 🞏 |
| Brug af skærmmedier i fritiden hjælper barnet med at lære at læse | 🞏 | 🞏 | 🞏 | 🞏 |
| Brug af skærmmedier i fritiden er en hjælp for barnet i forhold til at lære at regne | 🞏 | 🞏 | 🞏 | 🞏 |
| Jeg er bekymret for barnets skærmforbrug af hensyn til hans/hendes sundhed og udvikling | 🞏 | 🞏 | 🞏 | 🞏 |
| Jeg er bekymret for barnets skærmforbrug af hensyn til hans/hendes sociale liv | 🞏 | 🞏 | 🞏 | 🞏 |
| Barnet har svært ved at finde på noget at lave, hvis han/hun ikke må bruge skærmmedier | 🞏 | 🞏 | 🞏 | 🞏 |
| Barnet udtrykker et ønske om at bruge skærmmedier hver dag | 🞏 | 🞏 | 🞏 | 🞏 |
| Det giver ofte anledning til konflikt, hvis jeg prøver at begrænse barnets skærmtid | 🞏 | 🞏 | 🞏 | 🞏 |
| Den tid som mit barn bruger på skærmmedier, er overvejende af stillesiddende karakter | 🞏 | 🞏 | 🞏 | 🞏 |

## 17.1 Hvor gammel var barnet, da han/hun fik sin egen bærbare computer?

🞏 0 år

🞏 1 år

🞏 2 år

🞏 3 år

🞏 4 år

🞏 5 år

🞏 6 år

🞏 7 år

🞏 Barnet har ikke sin egen bærbare computer

**17.2 Hvor gammel var barnet, da han/hun fik sin egen stationære computer?**

🞏 0 år

🞏 1 år

🞏 2 år

🞏 3 år

🞏 4 år

🞏 5 år

🞏 6 år

🞏 7 år

🞏 Barnet har ikke sin egen stationære computer

## 17.3 Hvor gammel var barnet, da han/hun fik sin egen smartphone?

❑ 0 år

❑ 1 år

❑ 2 år

❑ 3 år

❑ 4 år

❑ 5 år

❑ 6 år

❑ 7 år

❑ Barnet har ikke fået sin egen smartphone

## 17.4 Hvor gammel var barnet, da han/hun fik sin egen tablet?

🞏 0 år

🞏 1 år

🞏 2 år

🞏 3 år

🞏 4 år

🞏 5 år

🞏 6 år

🞏 7 år

🞏 Barnet har ikke fået sin egen tablet

| **I de sidste spørgsmål vil vi gerne vide noget om dit skærmforbrug i forbindelse med dit arbejde og i fritiden.** |
| --- |

| **Dit eget skærmforbrug** |
| --- |

## 18. Er hjemmet din primære studie- eller arbejdsplads?

🞏 Ja

🞏 Nej

🞏 Jeg er hverken studerende eller i arbejde

**18.1 Inden for den seneste måned, hvor meget tid har du typisk brugt per dag på arbejdsrelaterede opgaver i hjemmet på skærmenheder?**
Svar både for hverdag og weekend

(OBS! Svar kun på dette spørgsmål ved ”JA” eller ”NEJ” til spørgsmål 18)

*Hverdag (tid per dag)*

🞏 Ingen

🞏 1-29 minutter

🞏 30-59 minutter

🞏 1-2 timer

🞏 2-3 timer

🞏 3-5 timer

🞏 5-7 timer

🞏 Mere end 7 timer

*Weekenddag (tid per dag)*

🞏 Ingen

🞏 1-29 minutter

🞏 30-59 minutter

🞏 1-2 timer

🞏 2-3 timer

🞏 3-5 timer

🞏 5-7 timer

🞏 Mere end 7 timer

**19. Inden for den seneste måned, hvor meget tid har du typisk brugt per dag på følgende skærmaktiviteter i fritiden?**
Svar både for hverdag og weekend.

|  | Hverdag (tid per dag) | | | | | | | | Weekenddag (tid per dag) | | | | | | | |
| --- | --- | --- | --- | --- | --- | --- | --- | --- | --- | --- | --- | --- | --- | --- | --- | --- |
|  | Ingen | 1-29 min | 30-59 min | 1-2 t | 2-3 t | 3-4 t | 4-5 t | 5 t el. mere | Ingen | 1-29 min | 30-59 min | 1-2 t | 2-3 t | 3-4 t | 4-5 t | 5 t el. mere |
| Film, TV-serier, Youtube film, underholdningsprogrammer | 🞏 | 🞏 | 🞏 | 🞏 | 🞏 | 🞏 | 🞏 | 🞏 | 🞏 | 🞏 | 🞏 | 🞏 | 🞏 | 🞏 | 🞏 | 🞏 |
| Spil (på smartphone, tablet, spilkonsol, PC) | 🞏 | 🞏 | 🞏 | 🞏 | 🞏 | 🞏 | 🞏 | 🞏 | 🞏 | 🞏 | 🞏 | 🞏 | 🞏 | 🞏 | 🞏 | 🞏 |
| Sociale medier eller andre kommunikationstyper (f.eks. Facebook, Messenger, Twitter, Whatsapp, Snapchat, Instagram, privat E-mail, SMS) | 🞏 | 🞏 | 🞏 | 🞏 | 🞏 | 🞏 | 🞏 | 🞏 | 🞏 | 🞏 | 🞏 | 🞏 | 🞏 | 🞏 | 🞏 | 🞏 |
| Videosamtale (f.eks. Facetime, Skype) | 🞏 | 🞏 | 🞏 | 🞏 | 🞏 | 🞏 | 🞏 | 🞏 | 🞏 | 🞏 | 🞏 | 🞏 | 🞏 | 🞏 | 🞏 | 🞏 |
| Surfe på nettet (f.eks. læse nyheder, handle, Google søgninger) | 🞏 | 🞏 | 🞏 | 🞏 | 🞏 | 🞏 | 🞏 | 🞏 | 🞏 | 🞏 | 🞏 | 🞏 | 🞏 | 🞏 | 🞏 | 🞏 |
| Andet (f.eks. foto- eller videoredigering, tegneprogrammer, tekstbehandling) | 🞏 | 🞏 | 🞏 | 🞏 | 🞏 | 🞏 | 🞏 | 🞏 | 🞏 | 🞏 | 🞏 | 🞏 | 🞏 | 🞏 | 🞏 | 🞏 |

Hvis du har svaret **andet**, giv eksempler på hvad: ________________________________________

| Tak for din deltagelse i undersøgelsen |
| --- |
