# Supplementary material for: The development of a questionnaire to assess leisure time screen-based media use and its proximal correlates in children (SCREENS-Q)
Source: BMC Public Health. 2020 May 12;20:664. doi: 10.1186/s12889-020-08810-6 (PMC7216486; doi:10.1186/s12889-020-08810-6)
Supplement: Supplementary file 2 — Additional file 2. Translated version of the SCREENS-Q in English [file 12889_2020_8810_MOESM2_ESM.docx]

| The purpose of the SCREENS questionnaire is to investigate the screen media use and behaviour of children.  We hope that you will answer the questionnaire with as much detail as possible, on behalf of your child. If the child lives with you only part time, then answer the questions based on the screen activities that take place when the child is with you.  **The questionnaire takes approximately 15 minutes to complete.** |
| --- |

## 1. What is your relationship with the child?

🞏 Father

🞏 Step-father

🞏 Mother

🞏 Step-mother

🞏 Other

If other, please specify:

________________________________________

**2. How many people live in your household?**
Also include those who only partially reside in the household, for example children over which you have joint custody.

_______

| The child's access to screen media. |
| --- |

**3. How many of the following screen media devices are present in the household where the child lives?**
Include all screen-media devices, including devices that belong to people who only partially reside in the household, and devices that the child does not have access to. Devices that are never used by anyone should not be included.

|  | Enter one of the answers for each screen media device | | | | | |
| --- | --- | --- | --- | --- | --- | --- |
|  | 0 | 1 | 2 | 3 | 4 | 5 or more |
| Laptop | 🞏 | 🞏 | 🞏 | 🞏 | 🞏 | 🞏 |
| Desktop computer | 🞏 | 🞏 | 🞏 | 🞏 | 🞏 | 🞏 |
| Tablet/iPad | 🞏 | 🞏 | 🞏 | 🞏 | 🞏 | 🞏 |
| Smartphone | 🞏 | 🞏 | 🞏 | 🞏 | 🞏 | 🞏 |
| Television | 🞏 | 🞏 | 🞏 | 🞏 | 🞏 | 🞏 |
| Non-handheld gaming console (e.g., Xbox, PlayStation, Nintendo) | 🞏 | 🞏 | 🞏 | 🞏 | 🞏 | 🞏 |
| Handheld gaming console (e.g., PSVita, PSP, Nintendo Switch, Gameboy) | 🞏 | 🞏 | 🞏 | 🞏 | 🞏 | 🞏 |
| E-reader (e-book reader) | 🞏 | 🞏 | 🞏 | 🞏 | 🞏 | 🞏 |
| Other | 🞏 | 🞏 | 🞏 | 🞏 | 🞏 | 🞏 |

If other, please specify:

________________________________________

**4. How often has the child used the following screen media devices in the household within the past month?**
Only include screen media devices belonging to the household.

|  | *Select one option per display device* | | | | |
| --- | --- | --- | --- | --- | --- |
|  | Every day, or almost every day of the week | 4-5 days a week | 2-3 days a week | 1 day or less per week | Never |
| Laptop | 🞏 | 🞏 | 🞏 | 🞏 | 🞏 |
| Desktop computer | 🞏 | 🞏 | 🞏 | 🞏 | 🞏 |
| Tablet/iPad | 🞏 | 🞏 | 🞏 | 🞏 | 🞏 |
| Smartphone | 🞏 | 🞏 | 🞏 | 🞏 | 🞏 |
| Television | 🞏 | 🞏 | 🞏 | 🞏 | 🞏 |
| Non-handheld gaming console (e.g., Xbox, PlayStation, Nintendo) | 🞏 | 🞏 | 🞏 | 🞏 | 🞏 |
| Handheld gaming console (e.g., PSVita, PSP, Nintendo Switch, Gameboy) | 🞏 | 🞏 | 🞏 | 🞏 | 🞏 |
| E-reader (e-book reader) | 🞏 | 🞏 | 🞏 | 🞏 | 🞏 |
| Other | 🞏 | 🞏 | 🞏 | 🞏 | 🞏 |

If other, please specify:

________________________________________

## 5. Indicate whether the child has their own of the following screen media devices.

|  | *Select one option per screen device* | |
| --- | --- | --- |
|  | Yes | No |
| Laptop | 🞏 | 🞏 |
| Desktop computer | 🞏 | 🞏 |
| Tablet/iPad | 🞏 | 🞏 |
| Smartphone | 🞏 | 🞏 |
| Television | 🞏 | 🞏 |
| Non-handheld gaming console (e.g., Xbox, PlayStation, Nintendo) | 🞏 | 🞏 |
| Handheld gaming console (e.g., PSVita, PSP, Nintendo Switch, Gameboy) | 🞏 | 🞏 |
| E-reader (e-book reader) | 🞏 | 🞏 |
| Other | 🞏 | 🞏 |

If other, please specify:

________________________________________

## 6. Does the child bring the following screen media devices to school?

| Select one option for each line | | | |  |
| --- | --- | --- | --- | --- |
| Yes, daily or almost daily | Yes, weekly | Yes, less often than weekly | No, never |  |
| Smartphone | 🞏 | 🞏 | 🞏 | 🞏 |
| Tablet | 🞏 | 🞏 | 🞏 | 🞏 |
| Laptop | 🞏 | 🞏 | 🞏 | 🞏 |
| Handheld gaming console (e.g., PSVita, PSP, Nintendo Switch, Gameboy) | 🞏 | 🞏 | 🞏 | 🞏 |

## 7. Does the child use a tablet, smartphone, or computer in connection with school-related activities?

🞏 Yes, daily

🞏 Yes, weekly

🞏 Yes, less often than weekly

🞏 No, never

🞏 Don't know

## 8. Does the child use a tablet, smartphone, or other screen media device in break time, for example to play a screen-based game?

**Do not include the use of smartphones to contact parents**

🞏 Yes, daily

🞏 Yes, weekly

🞏 Yes, less often than weekly

🞏 No, never

🞏 Don't know

## 8.1 Does the child use a tablet, smartphone, or other screen media device during after school care, for example to play a screen-based game?

**Do not include the use of smartphones to contact parents only**

🞏 Yes, daily

🞏 Yes, weekly

🞏 Yes, less often than weekly

🞏 No, never

🞏 Don't know

🞏 My child does not attend after school care

| **The use and content of the child's screen media.** |
| --- |

**9. Within the past month, how much time has the child typically spent per day on the following screen-based activities during leisure time?**
Put one cross mark in each line - both for a typical weekday and weekend day (mins = minutes, hrs = hours)

|  | Weekday (time per day) | | | | | | | | Weekend days (time per day) | | | | | | | |
| --- | --- | --- | --- | --- | --- | --- | --- | --- | --- | --- | --- | --- | --- | --- | --- | --- |
|  | None | 1-29 mins | 30-59 mins | 1-2 hrs | 2-3 hrs | 3-4 hrs | 4-5 hrs | 5 hrs or more | None | 1-29 mins | 30-59 mins | 1-2 hrs | 2-3 hrs | 3-4 hrs | 4-5 hrs | 5 hrs or more |
| Movies, TV shows, YouTube video clips/movies, entertainment programs | 🞏 | 🞏 | 🞏 | 🞏 | 🞏 | 🞏 | 🞏 | 🞏 | 🞏 | 🞏 | 🞏 | 🞏 | 🞏 | 🞏 | 🞏 | 🞏 |
| Games (on smartphone, tablet, game console, PC) | 🞏 | 🞏 | 🞏 | 🞏 | 🞏 | 🞏 | 🞏 | 🞏 | 🞏 | 🞏 | 🞏 | 🞏 | 🞏 | 🞏 | 🞏 | 🞏 |
| School-related tasks using screen media devices | 🞏 | 🞏 | 🞏 | 🞏 | 🞏 | 🞏 | 🞏 | 🞏 | 🞏 | 🞏 | 🞏 | 🞏 | 🞏 | 🞏 | 🞏 | 🞏 |
| Video calls (e.g., Facetime, Skype) | 🞏 | 🞏 | 🞏 | 🞏 | 🞏 | 🞏 | 🞏 | 🞏 | 🞏 | 🞏 | 🞏 | 🞏 | 🞏 | 🞏 | 🞏 | 🞏 |
| Social media or other types of communication (e.g.Facebook, Messenger, Twitter, WhatsApp, Snapchat, Instagram, Email, SMS) | 🞏 | 🞏 | 🞏 | 🞏 | 🞏 | 🞏 | 🞏 | 🞏 | 🞏 | 🞏 | 🞏 | 🞏 | 🞏 | 🞏 | 🞏 | 🞏 |
| Other (for example, drawing programs, making musical or stop-motion videos) | 🞏 | 🞏 | 🞏 | 🞏 | 🞏 | 🞏 | 🞏 | 🞏 | 🞏 | 🞏 | 🞏 | 🞏 | 🞏 | 🞏 | 🞏 | 🞏 |

If you selected **other**, please provide examples of what this includes: ________________________________________

| **Media behaviour in the home** |
| --- |

## 10. Does it ever happen that the television is on while you are not watching it?

🞏 Yes, daily or almost daily

🞏 Yes, weekly

🞏 Yes, less often than weekly

🞏 No, never

🞏 Don't have television in the household

| **Any rules for the child’s use of screen media** |
| --- |

## 11. Indicate whether you agree or disagree with the statements below regarding the child's screen media use at home:

|  | Agree | Disagree |
| --- | --- | --- |
| a. The child must always ask for permission before using screen media | 🞏 | 🞏 |
| b. There are fixed boundaries for how much time the child may use screen media | 🞏 | 🞏 |
| c. There are fixed boundaries for when the child may use screen media during the day | 🞏 | 🞏 |
| d. There are fixed boundaries for what games the child is allowed to play | 🞏 | 🞏 |
| e. There are fixed boundaries for what movies, YouTube clips, TV shows, and entertainment programs the child may watch | 🞏 | 🞏 |

| **The child’s screen media use during the day.** |
| --- |

**12. How many days in a typical week does the child use screen media in the following time periods?**
(e.g., Watching TV, playing games, doing school-related activities with screen media)
Put one cross mark in each time, both for weekdays and weekend days

|  | Number of weekdays per week | | | | | | Number of weekend days per week | | |
| --- | --- | --- | --- | --- | --- | --- | --- | --- | --- |
|  | 0 days | 1 day | 2 days | 3 days | 4 days | 5 days | 0 days | 1 day | 2 days |
| ...within half an hour after he/she wakes up in the morning? | 🞏 | 🞏 | 🞏 | 🞏 | 🞏 | 🞏 | 🞏 | 🞏 | 🞏 |
| ...within half an hour before he/she goes to sleep in the evening? | 🞏 | 🞏 | 🞏 | 🞏 | 🞏 | 🞏 | 🞏 | 🞏 | 🞏 |

13. How much time does the child spend on screen media during a typical day within the following periods? **Put one cross mark in each line (mins = minutes, hrs = hours):**

|  | None | 1-15 mins | 15-30 mins | 30-45 mins | 45-60 mins | 1-1½ hrs | 1½-2 hrs | 2-2½ hrs | 2½-3 hrs | 3-4 hrs | 4-5 hrs | More than 5 hrs |
| --- | --- | --- | --- | --- | --- | --- | --- | --- | --- | --- | --- | --- |
| Weekday (before school) | 🞏 | 🞏 | 🞏 | 🞏 | 🞏 | 🞏 | 🞏 | 🞏 | 🞏 | 🞏 | 🞏 | 🞏 |
| Weekday (after school, but before dinner) | 🞏 | 🞏 | 🞏 | 🞏 | 🞏 | 🞏 | 🞏 | 🞏 | 🞏 | 🞏 | 🞏 | 🞏 |
| Weekday (after dinner) | 🞏 | 🞏 | 🞏 | 🞏 | 🞏 | 🞏 | 🞏 | 🞏 | 🞏 | 🞏 | 🞏 | 🞏 |
| Weekend day (before 12 p.m.) | 🞏 | 🞏 | 🞏 | 🞏 | 🞏 | 🞏 | 🞏 | 🞏 | 🞏 | 🞏 | 🞏 | 🞏 |
| Weekend day (after 12 p.m., but before dinner) | 🞏 | 🞏 | 🞏 | 🞏 | 🞏 | 🞏 | 🞏 | 🞏 | 🞏 | 🞏 | 🞏 | 🞏 |
| Weekend day (after dinner) | 🞏 | 🞏 | 🞏 | 🞏 | 🞏 | 🞏 | 🞏 | 🞏 | 🞏 | 🞏 | 🞏 | 🞏 |

| **How the child uses screen media.** |
| --- |

**14. When the child uses screen media, how often does he/she use more than one screen media device at a time?**
(For example, watching TV and using a tablet at the same time)

🞏 Never

🞏 Rarely

🞏 Sometimes

🞏 Often

🞏 Always

**15. When the child uses screen media, is it usually...**
Put only one cross mark

🞏 ...with you/other adults?

🞏 ...together with friends?

🞏 ...together with siblings?

🞏 ...alone?

16. Below is a list of statements about the child's use of screen media. **Please indicate how much you agree with each statement.**

|  | Strongly agree | Partly agree | Partly disagree | Strongly disagree |
| --- | --- | --- | --- | --- |
| If the child is given the choice he/she will almost always choose to spend his/her time on screen-based activities | 🞏 | 🞏 | 🞏 | 🞏 |
| If the child is given the choice, he/she will almost always choose to spend his/her time playing without a screen | 🞏 | 🞏 | 🞏 | 🞏 |
| The use of screen media enhances the child's sense of community with other children | 🞏 | 🞏 | 🞏 | 🞏 |
| The use of screen media often helps the child calm down | 🞏 | 🞏 | 🞏 | 🞏 |
| The child and I often use screen media together | 🞏 | 🞏 | 🞏 | 🞏 |
| The child's use of screen media results in many pleasant conversations with the child | 🞏 | 🞏 | 🞏 | 🞏 |
| The child's use of screen media enhances his/her creativity and imagination | 🞏 | 🞏 | 🞏 | 🞏 |
| The child has an appropriate amount of screen time in his/her leisure time | 🞏 | 🞏 | 🞏 | 🞏 |
| Using screen media during leisure time helps the child learn how to write and spell | 🞏 | 🞏 | 🞏 | 🞏 |
| Using screen media during leisure time helps the child learn to read | 🞏 | 🞏 | 🞏 | 🞏 |
| Using screen media during leisure time helps the child learn how to calculate | 🞏 | 🞏 | 🞏 | 🞏 |
| I am concerned about the child's screen media use in relation to his/her health and development | 🞏 | 🞏 | 🞏 | 🞏 |
| I am concerned about the child's screen media use for the sake of his/her social life | 🞏 | 🞏 | 🞏 | 🞏 |
| The child has difficulty in thinking of things to do if he/she is not allowed to use screen media | 🞏 | 🞏 | 🞏 | 🞏 |
| The child expresses a desire to use screen media every day | 🞏 | 🞏 | 🞏 | 🞏 |
| It often causes conflict if I try to limit the child’s screen media use | 🞏 | 🞏 | 🞏 | 🞏 |
| The amount of time my child spends on screen media is predominately sedentary | 🞏 | 🞏 | 🞏 | 🞏 |

## 17.1 How old was the child when he/she got his/her own laptop?

🞏 0 years

🞏 1 year

🞏 2 years

🞏 3 years

🞏 4 years

🞏 5 years

🞏 6 years

🞏 7 years

🞏 The child does not have their own laptop

**17.2 How old was the child when he/she got his/her own desktop computer?**

🞏 0 years

🞏 1 year

🞏 2 years

🞏 3 years

🞏 4 years

🞏 5 years

🞏 6 years

🞏 7 years

🞏 The child does not have his/her own desktop computer

## 17.3 How old was the child when he/she got his/her own smart phone?

❑ 0 years

❑ 1 year

❑ 2 years

❑ 3 years

❑ 4 years

❑ 5 years

❑ 6 years

❑ 7 years

❑ The child does not have his/her own smart phone

## 17.4 How old was the child when he/she got his/her own tablet?

🞏 0 years

🞏 1 year

🞏 2 years

🞏 3 years

🞏 4 years

🞏 5 years

🞏 6 years

🞏 7 years

🞏 The child does not have his/her own tablet

| **In the last questions, we would like to know something about your own screen media use in relation to your work and leisure time.** |
| --- |

| **Your own screen media use** |
| --- |

## 18. Is your home your primary place of study or work?

🞏 Yes

🞏 No

🞏 I am neither working nor studying

**18.1 In the past month, how much time have you typically spent per day on work-related tasks at home on screen media devices?**
Put one cross mark for weekdays and one for weekend days

(Note: Only answer this question if you answered “YES” or “NO” to question 18)

*Weekday (time per day)*

🞏 None

🞏 1-29 mins

🞏 30-59 mins

🞏 1-2 hrs

🞏 2-3 hrs

🞏 3-5 hrs

🞏 5-7 hrs

🞏 More than 7 hrs

*Weekend days (time per day)*

🞏 None

🞏 1-29 mins

🞏 30-59 mins

🞏 1-2 hrs

🞏 2-3 hrs

🞏 3-5 hrs

🞏 5-7 hrs

🞏 More than 7 hrs

**19. Within the past month, how much time have you typically spent per day on the following screen-based activities during leisure time**
Put one cross mark in each line - both for a typical weekday and weekend day (mins = minutes, hrs = hours)

|  | Weekday (time per day) | | | | | | | | Weekend days (time per day) | | | | | | | |
| --- | --- | --- | --- | --- | --- | --- | --- | --- | --- | --- | --- | --- | --- | --- | --- | --- |
|  | None | 1-29 mins | 30-59 mins | 1-2 hrs | 2-3 hrs | 3-4 hrs | 4-5 hrs | 5 hrs or more | None | 1-29 mins | 30-59 mins | 1-2 hrs | 2-3 hrs | 3-4 hrs | 4-5 hrs | 5 hrs or more |
| Movies, TV shows, YouTube clips and movies, entertainment programs | 🞏 | 🞏 | 🞏 | 🞏 | 🞏 | 🞏 | 🞏 | 🞏 | 🞏 | 🞏 | 🞏 | 🞏 | 🞏 | 🞏 | 🞏 | 🞏 |
| Games (on smartphone, tablet, game console, PC) | 🞏 | 🞏 | 🞏 | 🞏 | 🞏 | 🞏 | 🞏 | 🞏 | 🞏 | 🞏 | 🞏 | 🞏 | 🞏 | 🞏 | 🞏 | 🞏 |
| Social media, or other forms of communication (e.g., Facebook, Messenger, Twitter, WhatsApp, Snapchat, Instagram, private email, SMS) | 🞏 | 🞏 | 🞏 | 🞏 | 🞏 | 🞏 | 🞏 | 🞏 | 🞏 | 🞏 | 🞏 | 🞏 | 🞏 | 🞏 | 🞏 | 🞏 |
| Video calls (e.g., Facetime, Skype) | 🞏 | 🞏 | 🞏 | 🞏 | 🞏 | 🞏 | 🞏 | 🞏 | 🞏 | 🞏 | 🞏 | 🞏 | 🞏 | 🞏 | 🞏 | 🞏 |
| Surfing the web (e.g., reading the news, shopping, Google searches) | 🞏 | 🞏 | 🞏 | 🞏 | 🞏 | 🞏 | 🞏 | 🞏 | 🞏 | 🞏 | 🞏 | 🞏 | 🞏 | 🞏 | 🞏 | 🞏 |
| Other (e.g., photo or video editing, drawing programs, word processing) | 🞏 | 🞏 | 🞏 | 🞏 | 🞏 | 🞏 | 🞏 | 🞏 | 🞏 | 🞏 | 🞏 | 🞏 | 🞏 | 🞏 | 🞏 | 🞏 |

If you selected **other**, please provide an example of what this includes: ________________________________________

| Thank you for your participation |
| --- |
